# Particulate Matter–Induced Health Effects: Who Is Susceptible?

**DOI:** 10.1289/ehp.1103846

**Published:** 2011-07-01

**Authors:** Ken-ichiro Inoue, Hirohisa Takano

**Affiliations:** Department of Public Health and Molecular Toxicology, School of Pharmacy, Kitasato University, Tokyo, Japan, E-mail: inouek@pharm.kitasato-u.ac.jp; Environmental Health Sciences Division National Institute for Environmental Studies, Kitasato University, Tsukuba, Japan

We read with great interest a recent review by Sacks et al. (2011) and would like to add some comments to facilitating effects of particulate matter (PM) on preexisting respiratory diseases. First of all, the adverse effects of PM/diesel exhaust particles (DEP) on chronic obstructive pulmonary disease (COPD) pathophysiology seem to be controversial. Subjects with pulmonary emphysema are epidemiologically susceptible to PM (Dockery et al. 1993; Euler et al. 1987; MacNee and Donaldson 2003; Thishan Dharshana and Coowanitwong 2008). Further, as noted by Sacks et al. (2011), Lopes et al. (2009) have experimentally shown that chronic (2 months) exposure to an ambient level (mean concentration, 34 µg/m^3^) of PM_10_ (PM < 10 µm in aerodynamic diameter) worsens murine emphysema induced by papain. In contrast, in our previous study (Inoue et al. 2010) we did not obtain apparent evidence that a single intratracheal administration of DEP [200 µg/animal, a dose high enough to worsen infectious lung injury (Takano et al. 2002)] exacerbates porcine pancreatic elastase–elicited pulmonary emphysema in mice. Possible explanations for this opposite phenomenon may include differences in animal strains or species, pathological conditions (type and/or  degree of emphysematous inflammation), and/or DEP exposure protocols (route, dose, timing, duration, and/or terminal point). Additional in-depth studies will be required to conclude PM/DEP has adverse effects on COPD pathophysiology.

Secondly, from a biological point of view, pulmonary fibrosis (PF) should be added to the list of PM-susceptible respiratory diseases. Recently, Decologne et al. (2010) showed that exposure to carbon black nanoparticles exacerbates bleomycin-induced PF in mice. More recently, we demonstrated that a single intratracheal instillation of tiny carbon black nanoparticles (14 nm) at a dose of 10 µg/mouse aggravates PF, suggesting that exposure to trace amounts of PM can exacerbate pathophysiology (Kamata et al. 2011). Accordingly, careful attention should be paid to PF patients who are at risk of environmental and occupational exposure to PM, although further basic and clinical research is necessary.
